# An international consensus on screening and monitoring early‐stage type 1 diabetes: A roadmap to European implementation

**DOI:** 10.1111/dom.70569

**Published:** 2026-03-12

**Authors:** Sufyan Hussain, Timothy Tree, Chantal Mathieu, Tomasz Klupa, Anna‐Kaisa Tuomaala, Maartje de Wit, Olga Kordonouri, Katarina Braune, Jaivir Pall, Luis Castano, Rachel E. J. Besser, Júlia Galhardo, Francesca Ulivi, Emanuele Bosi, Uroš Bogdanovic, Coralie Alabert, Tadej Battelino

**Affiliations:** ^1^ Department of Diabetes, School of Cardiovascular, Metabolic Medicine and Sciences King's College London London UK; ^2^ Department of Diabetes and Endocrinology Guy's & St Thomas' NHS Foundation Trust London UK; ^3^ Institute of Diabetes, Endocrinology and Obesity King's Health Partners London UK; ^4^ Department of Immunobiology, School of Immunology and Microbial Sciences King's College London London UK; ^5^ National Institute for Health Research Biomedical Research Centre, Guy's and St. Thomas' NHS Foundation Trust and Kings College London London UK; ^6^ Clinical and Experimental Endocrinology University of Leuven Leuven Belgium; ^7^ Department of Metabolic Diseases, Centre for Advanced Technologies in Diabetes Jagiellonian University Medical College Krakow Poland; ^8^ Department of Metabolic Diseases, Psychodiabetology Unit Jagiellonian University Medical College Krakow Poland; ^9^ Department of Metabolic Diseases and Diabetology University Hospital in Kraków Krakow Poland; ^10^ Pediatric Research Center Helsinki University Children's Hospital Helsinki Finland; ^11^ Faculty of Medicine University of Helsinki Helsinki Finland; ^12^ Medical Psychology, Amsterdam UMC, Location Vrije Universiteit Amsterdam Amsterdam the Netherlands; ^13^ Mental Health, Amsterdam Public Health Amsterdam the Netherlands; ^14^ Kinder‐ und Jugendkrankenhaus auf der Bult Hannover Germany; ^15^ Hasso Plattner Institute for Digital Engineering University of Potsdam Potsdam Germany; ^16^ Institute of Medical Informatics, Charité ‐ Universitätsmedizin Berlin Germany; ^17^ INNODIA Patient Advisory Committee Madrid Spain; ^18^ Hospital Universitario Cruces, Universidad del País Vasco, IIS Biobizcaia, CIBERDEN, CIBERER, Endo‐ERN Baracaldo Spain; ^19^ Centre for Human Genetics, Nuffield Department of Medicine, NIHR Oxford Biomedical Research Centre University of Oxford Oxford UK; ^20^ Department of Paediatrics John Radcliffe Hospital Oxford UK; ^21^ Dona Estefânia Hospital—ULSSJosé Lisbon Portugal; ^22^ Fondazione Italiana Diabete Milan Italy; ^23^ Diabetes Research Institute, IRCCS San Raffaele Hospital, and San Raffaele Vita Salute University Milan Italy; ^24^ IDF Europe YOURAH Network Belgrade Serbia; ^25^ IDF Europe YOURAH Network Lille France; ^26^ Department of Endocrinology, Diabetes, and Metabolic Diseases University Children's Hospital, University Medical Centre Ljubljana Ljubljana Slovenia; ^27^ Faculty of Medicine University of Ljubljana Ljubljana Slovenia

**Keywords:** autoimmunity, beta cell function, glycaemic control, health economics, islets, type 1 diabetes

## Abstract

Type 1 diabetes (T1D) is a chronic autoimmune disease that results in loss of insulin‐secreting pancreatic β‐cells in the islets of Langerhans. A diagnosis of T1D is typically associated with children and adolescents, yet half of all diagnoses of T1D are made in adults. In children and adolescents, T1D is often first recognized following hospitalization for diabetic ketoacidosis (DKA), which occurs in approximately 20%–50% of new‐onset T1D for people younger than 18 years of age in Europe. For adults with new‐onset T1D, DKA rates of up to 24% are estimated. Early‐stage T1D, during the asymptomatic period, can be detected through screening for multiple islet autoantibodies in blood samples, including capillary and venous samples, and such programs are made more popular by the availability of disease‐modifying therapies for early‐stage T1D. For individuals who screen positive for early‐stage T1D, participation in monitoring programs can greatly reduce the incidence of DKA once symptomatic hyperglycemia develops, as well as reducing severity of symptoms of T1D at onset. Education and awareness of the clinically relevant features of symptomatic T1D can also support the psychological wellbeing of people with early‐stage T1D and minimize distress at the point when insulin treatment is necessary. All of these consequences come with a predicted reduced burden of healthcare costs for managing T1D at a population level, and general population screening for islet autoantibodies is underway. In this European perspective, we discuss the imperatives and the components of implementation of general population screening for early‐stage T1D.

## 1 INTRODUCTION AND RATIONALE

Type 1 diabetes (T1D) is a chronic disease, characterized by hyperglycemia following progressive autoimmune β‐cell destruction and consequent lack of insulin.^1^ T1D affects around 2.8 million people across Europe, predicted to increase to 3.9 million persons by 2040.^2^ Data for 1989–2013 from 20 EURODIAB centers show that incidence rates vary considerably between European countries,^3^, ^4^ with reported standardized rates per 100 000 individuals as high as 60.9 in Finland and 31.8 in Sweden, to 11.6 in Slovenia and 13.8 in Austria. In all countries, incidence rates showed an annual increase of 1.9–6.6% from 1989 onwards.^3^ A separate EURODIAB report covering trends across 22 countries estimated a pooled annual rate of increase in incidence of T1D of 3.4% across Europe.^4^ Overall incidence rates of 20.96 per 100 000 for 2022 have been estimated from a systematic review and meta‐analysis.^5^ Since T1D is more commonly diagnosed in children, the incidence of T1D in children under 15 years of age in Europe is predicted to double within 20 years.^4^

Living with T1D confers a threefold excess risk for cardiovascular disease (CVD) and twofold excess risk of mortality.^6^ The risks for microvascular complications, such as retinopathy, neuropathy and nephropathy, are also well established.^7^ The high occurrence of diabetic ketoacidosis (DKA) at diagnosis is associated with persistent cognitive impairment.^8^ Although intensive glycemic management with insulin is proven to significantly reduce the risks of long‐term complications of T1D,^9^ more than 60% of children and adolescents in international diabetes registries did not achieve recommended HbA1c targets of <53 mmol/mol (<7.0%) in 2022,^10^ which can increase to 85% where recommended HbA1c targets are <48 mmol/mol (<6.5%).^11^ The challenge of achieving an optimal HbA1c target is highlighted by the CLOuD study, in which young people aged 10–16 years with newly diagnosed T1D (within the previous 21 days) were allocated to treatment with hybrid closed loop (HCL) insulin therapy.^12^ Although HCL therapy was associated with a 10 mmol/mol (0.9%) reduction in HbA1c over 48 months, compared to a control group on standard insulin therapy with multiple daily insulin (MDI) injections or insulin pump therapy, only 34% of HCL users achieved the <48 mmol/mol (<6.5%) target, despite access to the latest diabetes technology.

A public health initiative aimed at reducing both the human and economic impact of T1D is general population screening.^13^ There is a recognizable early stage of T1D, during which clinical symptoms of hyperglycemia are absent or undetectable.^14^ During this period, autoantibodies to pancreatic islet antigens can be detected, opening the door for a range of interventions that may significantly lessen both immediate and long‐term consequences.^15^ These include education and awareness initiatives, participation in monitoring programs and intervention studies, and the application of disease‐modifying therapy.^16^ Currently, awareness and management of early‐stage T1D have largely been restricted to individuals who have been screened for genetic susceptibility, or who are first or second‐degree relatives of a family member with a confirmed diagnosis of T1D. However, over 85% of people newly diagnosed with T1D have no family history or known predisposing genetic markers.^17^, ^18^ A number of initiatives in Europe and in the United States (US) have proven that general population screening for islet autoantibodies has significant positive outcomes for those who test positive.^19^, ^20^, ^21^ The focus of this consensus document is systematic general population screening to identify individuals with islet autoantibodies diagnostic for early‐stage T1D, independent of predisposing genetic risks.

The principles that underpin the value of public health screening for significant health conditions have been developed by the World Health Organization (WHO),^22^ and are also commonly referred to as the Wilson and Jungner criteria (Table 1). Many of these criteria have been met for the application of general population screening for islet autoantibodies to detect early‐stage T1D. In this context, the goals of general population screening for islet autoantibodies include: reducing the occurrence of DKA at diagnosis, improving outcomes across all stages of T1D, reducing healthcare costs associated with DKA and management of T1D, providing access to disease‐modifying therapy as early as possible (where available), and enabling participation in monitoring programs and clinical trials.^23^

**TABLE 1:** Wilson and Jungner criteria for public health screening for disease.^22^, ^139^

The condition should be an important public health problem There should be an accepted treatment for individuals who screen positive There should be a recognizable latent or early‐stage, with or without symptoms The target population should be clearly defined and able to be reached Facilities for diagnosis and monitoring should be available The pathophysiology of the condition should be understood, including the progression from latent/early to symptomatic stages of disease There should be a suitable test for detection, which is sensitive and specific at a population level The test should be acceptable to the screened population (i.e., have a low burden of participation) The test outcomes from the screening event for each individual should be clearly interpretable There should be an agreed policy on whom to treat The cost of screening and case‐finding (including costs for monitoring and treatment) should be economically balanced by the savings against possible costs of treating overt disease. Case‐finding should be a continuing process and not a once‐and‐for‐all effort.

In this consensus opinion, we summarize the rationale for wider general population screening for islet autoantibodies and review the opportunities and challenges that accompany such a public health initiative. The operational aspects of general population islet autoantibody screening have been described in detail in a separate international consensus^24^ and are not covered here. Drawing on examples of ongoing general population screening initiatives for early‐stage T1D^19^, ^25^, ^26^, ^27^ we identify the essential components of an effective screening program that can be implemented nationally across diverse healthcare systems. Importantly, we issue a call to action for healthcare and governmental stakeholders to more effectively advocate for this important unmet public health need.

## 2 INCIDENCE AND BURDEN OF DKA AT DIAGNOSIS OF TYPE 1 DIABETES

A key concern is that, across European healthcare services, a diagnosis of T1D is often made when a person is taken to the emergency room or admitted to hospital with DKA. The reported incidence of DKA at diagnosis of T1D in people who have not previously been aware of their islet autoantibody status varies widely across Europe,^28^, ^29^, ^30^, ^31^, ^32^ from as low as 20% in Scandinavian countries to 67% in Romania (Table 2), with significant regional variation. Incidence of DKA at diagnosis is higher among young children aged <2 years,^33^ and is also associated with higher regional deprivation indices.^32^, ^34^ Taken as a whole, the incidence of DKA at diagnosis for children and adolescents in Europe is estimated by the EURODIAB ACE study group at 40–42%.^29^ Importantly, incidence of DKA at diagnosis of T1D is increasing,^21^, ^28^, ^35^ independent of the increased prevalence reported during the coronavirus disease (COVID‐19) pandemic.^36^ For adults aged 18–45 years, the INNODIA Natural History Study has reported a DKA incidence on diagnosis of T1D of 23% across 18 diabetes clinical centers across Europe,^37^ which is consistent with the rate of 19–24% for adults aged 25–40 years reported by the T1D Exchange registry.^38^

**TABLE 2:** Rate of DKA at diagnosis of T1D in children and adolescents in European countries.

Country	% DKA	Year^a^	Number in study	Notes
Romania	67.0^29^	2001	21	EURODIAB questionnaire survey of diabetes centers
Poland	54.2^29^	2001	59	EURODIAB questionnaire survey of diabetes centers
	38.0^140^	2003	158	Single center retrospective observational study
	32.9^141^	2009	474	Regional retrospective observational study
	26.0^142^	2011	187	Single center retrospective observational study
	30.1^143^	2014	652	Single center retrospective observational study
	55.0^144^	2003	106	Single center retrospective observational study
	33.0^145^	2007	186	Single center retrospective observational study
Hungary	50.0^29^	2001	128	EURODIAB questionnaire survey of diabetes centers
	23.0^146^	1997		Questionnaire based single center survey
Lithuania	41.4^29^	2001	58	EURODIAB questionnaire survey of diabetes centers
	34.6^147^	2002		Retrospective observational study
Bulgaria	39.0^29^	2001	44	EURODIAB questionnaire survey of diabetes centres
	35.3^148^	1996	1248	Single center retrospective observational study
Austria	34.0^29^	2001	140	EURODIAB questionnaire survey of diabetes centers
	37.2^149^	2010	3331	Prospective population‐based incidence study
	37.7^28^	2020	1504	Retrospective observational study of clinical data
	37.5^115^	2013	4038	Retrospective registry study
	26.4^150^	2024	14 292	DPV Registry study inc. Germany, Switzerland, Luxembourg
Slovak Republic	35.6^29^	2001	109	EURODIAB questionnaire survey of diabetes centers
Slovenia	28.6^29^	2001	21	EURODIAB questionnaire survey of diabetes centers
	37.6^32^	2024	306	Registry‐based study of regional deprivation on DKA rates
	39.9^28^	2020	471	Retrospective observational study of clinical data
Netherlands	28.6^29^	2001	53	EURODIAB questionnaire survey of diabetes centers
Iceland	30.0^29^	2001	10	EURODIAB questionnaire survey of diabetes centers
	36.0^151^	2014	14	Cross‐sectional registry study
Germany	25.6^29^	2001	46	EURODIAB questionnaire survey of diabetes centers
	32.5^32^	2024	13 561	Registry‐based study of regional deprivation on DKA rates
	26.8^28^	2020	19 127	Retrospective observational study of clinical data
	28.3^117^	2020	127	Regional assessment of diabetes awareness campaign
	19.8^152^	2021	41 189	DPV Registry, Germany only 2000–2019.
	24.5^153^	2020	503	DPV Registry data for March–May 2019 (pre‐COVID‐19)
	44.7^153^	2020	532	DPV Registry data for March–May 2020 (COVID‐19)
	26.4^154^	2024	14 292	DPV Registry study inc. Austria, Switzerland, Luxembourg
United Kingdom	25.0^155^	2014	261	Retrospective observational study
	39.8^156^	2015	88	Questionnaire‐based regional study
	38.5^157^	2020	7378	National Paediatric Diabetes Audit, 2015–2020
France	54.0^158^	2003	72	Prospective clinical study
	43.9^159^	2014	1299	Retrospective observational study
Türkiye	29.0^160^	2001	62	Single center retrospective observational study
	50.8^161^	2014	354	Regional retrospective observational study
	44.2^121^	2013	401	Single center retrospective observational study
Spain	44.0^162^	1996	125	Single center retrospective observational study
	39.5^163^	2012	1169	Retrospective observational study
	38.6^164^	2023	267	Regional retrospective observational study
Sweden	19.5^28^	2020	6457	Retrospective observational study
	25.9^165^	2020	4167	National Diabetes Register, Annual Report 2020
Finland	22.4^166^	2007	585	Single center retrospective observational study
	19.4^167^	2010	1656	Retrospective registry study
	19.2^168^	2011	1518	Retrospective registry study
Italy	36.7^112^	2024	738	Initial outcomes from D1Ce pilot study
	42.5^32^	2024	4659	Registry‐based study of regional deprivation on DKA rates
	41.2^28^	2020	10 317	Retrospective observational study
	56.0^169^	2016	230	Retrospective observational study
Russia	30.0^170^	2008	2031	Regional (Moscow) retrospective study
Ireland	25.0^171^	2005	283	Prospective observational study
Portugal	48.4^172^	2024	574	Registry study during COVID‐19, DKA rate not influenced
Wales	35.2^32^	2024	769	Registry‐based study of regional deprivation on DKA rates
	25.0^28^	2020	1673	Retrospective observational study
Czechia	28.6^28^	2020	2261	Retrospective observational study
Denmark	20.8^28^	2020	3084	Retrospective observational study
	14.7^173^	2013	129	Prospective observational study
	17.9^49^	2013	2964	Retrospective registry study
	20.0^151^	2014	283	Cross‐sectional registry study
Luxembourg	43.8^28^	2020	192	Retrospective observational study of clinical data
	26.4^150^	2024	14 292	DPV Registry study inc. Germany, Switzerland, Austria
Norway	22.1^28^	2020	3331	Retrospective observational study of clinical data
	22.0^151^	2014	325	Cross‐sectional registry study
Belgium	25.6^174^	2014	242	Retrospective single center study
Israel	33.7^175^	2013	406	Retrospective observational study
	42.0^176^	2015	81	Single center observational study of ultraorthodox children
Malta	41.0^177^	2012	81	Prospective single center study
Serbia	32.9^178^	2013	720	Single center observational study

*Note*: The table shows the rates of DKA at diagnosis for children and adolescents <18 years of age reported in diverse studies as indicated.

^a^ The year refers to date of study publication, and the data are not longitudinal.

For individuals with new‐onset T1D, DKA results from reduced insulin activity due to partial or complete insulin deficiency. This is associated with increases in counter‐regulatory hormones, which promote gluconeogenesis in the liver without significant glucose uptake in peripheral tissues, leading to hyperglycemia.^39^, ^40^ Reduced glucose utilization and low insulin concentrations lead to lipolysis of endogenous triglycerides, resulting in high concentrations of free fatty acids that are oxidized in the liver to ketone bodies. At the same time, catabolism of muscle protein occurs, releasing amino acids that are both gluconeogenic and ketogenic. At presentation with DKA, people with newly diagnosed T1D exhibit the diagnostic triad of hyperglycemia, ketonemia, and metabolic acidosis.^41^, ^42^

DKA at onset of T1D is a potentially life‐threatening event, with both acute and chronic consequences. Cerebral edema occurs in 1.2% DKA cases at diagnosis,^43^ with high mortality in this small group (24%),^43^ making it the most common cause of DKA‐related fatality. Children and adolescents presenting with DKA often have significant circulatory volume depletion, which contributes to the development of acute kidney injury (AKI) at diagnosis. A single‐center study from Canada^44^ reported that 64.2% of DKA cases in children and adolescents aged <18 years were complicated by AKI. A second study from Italy^45^ reported an AKI incidence of 65.2% among children with DKA at diagnosis of T1D, all with confirmed renal tubular damage, and acute tubular necrosis in about a third.

Evidence also links DKA at onset of T1D with longer‐term challenges in metabolic control, including increased risk of subsequent episodes of DKA.^46^, ^47^ Diminished residual β‐cell function is also associated with DKA at diagnosis,^37^, ^48^ which can negatively affect long‐term HbA1c. In cross‐sectional data, moderate to severe DKA at diagnosis of T1D has been associated with a significantly higher HbA1c over time,^49^, ^50^, ^51^ compared to people with no or mild DKA at diagnosis. Similarly, although baseline HbA1c for children and adolescents with and without DKA at diagnosis converges within 12 months, the subsequent year‐on‐year rate of change in HbA1c was 0.16% higher for those with DKA.^52^

Neurocognitive function is also notably affected by episodes of DKA at diagnosis. Cerebral white matter volume is increased in children with DKA at onset of T1D,^53^ with higher mean diffusivity in the frontal, temporal, and parietal white matter. Although these morphological changes resolved, they were associated with poorer long‐term attention and memory scores, and a history of DKA in childhood is linked to disrupted memory.^54^ Increased total and regional white and grey matter volume for children aged 4 to <10 years with moderate or severe DKA at diagnosis has also been associated with reduced intelligence quotient (IQ) scores over 18 months, as well as reduced attention performance, compared to age‐ and HbA1c‐matched children with T1D without DKA.^55^ Similar impacts on IQ scores have also been reported for children aged 3–5 years with DKA at diagnosis, regardless of severity, compared to those without.^8^

Other reported acute and chronic consequences of DKA for new‐onset T1D include hypercoagulability leading to stroke or deep vein thrombosis, rhabdomyolysis, pulmonary and gastrointestinal complications^56^ although these are considered rare.

## 3 IDENTIFYING EARLY‐STAGE TYPE 1 DIABETES

**FIGURE 1:**
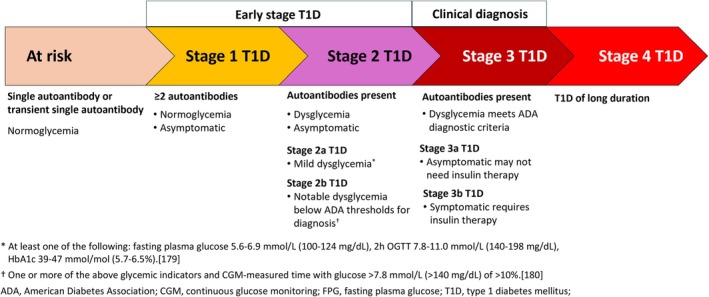
Stages of type 1 diabetes associated with a positive islet autoantibody screen. *At least one of the following: Fasting plasma glucose 5.6–6.9 mmol/L (100–124 mg/dL), 2h OGTT 7.8–11.0 mmol/L (140–198 mg/dL), HbA1c 39–47 mmol/mol (5.7–6.5%).^179^ ^†^One or more of the above glycemic indicators and CGM‐measured time with glucose >7.8 mmol/L (>140 mg/dL) of >10%.^180^ ADA, American Diabetes Association; CGM, continuous glucose monitoring; FPG, fasting plasma glucose; T1D, type 1 diabetes mellitus,

Established terminology defines three distinct stages of T1D that precede the requirement for insulin therapy, with progressive development of glycemic dysregulation and loss of glucose homeostasis (Figure 1).^1^, ^57^, ^58^ A critical part of this staging is the acknowledgement that each stage is a distinct part of the chronic autoimmune disease that is T1D, whether there are overt symptoms of hyperglycemia or not. The presymptomatic phase of T1D starts as Stage 1, characterized by the detection of two or more islet autoantibodies (from GAD65, insulin, IA2 and ZnT8), in the context of normoglycemia.^14^ Dysglycemia is indicative of Stage 2 T1D, whereas Stage 3 T1D meets the criteria for diagnosis of T1D as defined in the American Diabetes Association (ADA) standards of care, based on persistent hyperglycemia, with or without symptoms.^1^ Individuals identified in Stage 1 or Stage 2 T1D have a very high risk of progression to Stage 3 T1D, approaching 100% in children and adolescents.^59^ People in confirmed Stage 1 or Stage 2 T1D, after confirmation of their autoantibody status, should be encouraged to participate in a follow‐up monitoring program including education, glucose monitoring and psychological support, aiming at a timely and smooth transition to insulin therapy to prevent DKA and associated complications.^16^, ^20^, ^60^, ^61^, ^62^

Where approved, application of the disease‐modifying agent teplizumab (see below) is indicated for use in Stage 2 T1D, to delay onset of Stage 3 T1D.^63^ An objective for general population screening is to identify as many individuals with islet autoantibodies as possible, in order that progression to symptomatic Stage 3 T1D is reduced for as many individuals as possible, for as long as possible. Individuals identified and confirmed with a single islet autoantibody are considered to be at risk of T1D and this screening result requires careful interpretation. Figure 1 identifies this as an undefined observation, but in a general population screening program it is important to know the necessary messaging that should be conveyed to the screened individual. Both for children and adults, established guidelines indicate that either a single or a multiple autoantibody test result should be confirmed in a second sample,^16^, ^24^ using two independent methods,^64^ whilst also confirming negative status for other islet autoantibodies. In case of confirmation of a single autoantibody, the risk of progression to Stage 3 T1D is lower than for Stage 1 and Stage 2 T1D, but still carries a 10–15% risk over 15 years compared to 0.3% for the general population.^59^ The advice for future autoantibody retesting and glucose monitoring is less stringent than in individuals with multiple autoantibodies.^16^ Information on disease progression in adults is more limited than for children and adolescents, but specific recommendations for follow up and monitoring are available.^16^

## 4 POTENTIAL FOR IMMUNOTHERAPY OF EARLY‐STAGE T1D

The availability of disease‐modifying therapies for people with early‐stage T1D is an important reason to undertake general population screening. Teplizumab targets the thymus‐derived lymphocytes (T‐cells) that drive immune and inflammatory responses, including autoimmune reactions.^15^ For people with Stage 2 T1D, a single 14‐day course of teplizumab has been shown in a randomized controlled trial (RCT) to retard progression to Stage 3 T1D (48.4 vs. 24.4 months; hazard ratio [HR] = 0.41).^65^ Stage 3 T1D was diagnosed in 43% of the teplizumab intervention group, compared with 72% in the placebo group. In an extended (median 923 days) follow‐up,^66^ median time to diagnosis of Stage 3 T1D was 59.6 vs. 27.1 months for the intervention and placebo groups, respectively (HR = 0.46). Over the extended period, 50% of teplizumab‐treated participants remained diabetes‐free compared to 22% of the placebo arm. Treatment with teplizumab also improved β‐cell function compared to baseline on study entry, as measured by increased C‐peptide levels, whereas β‐cell function continued to decline in the placebo group. The changes in C‐peptide following teplizumab treatment were associated with reduced T‐cell secretion of the inflammatory cytokines interferon γ (IFNγ) and tumour necrosis factor‐α (TNFα).^66^

The US food and drug administration (FDA) approved teplizumab in November 2022 for the treatment of individuals with early‐stage T1D (stage 2),^67^ and has subsequently been approved by Health Canada,^68^ the United Kingdom Medicines and Healthcare products Regulatory agency (MHRA)^69^ and the European Medicines agency (EMA).^70^ Teplizumab has also been accepted for expedited review in the US for treatment of Stage 3 T1D through the FDA National Priority Voucher pilot program.^71^ Other immunotherapies are under active investigation for treatment of early‐stage T1D or newly diagnosed T1D. These include low‐dose antithymocyte globulin (ATG), which has been shown in clinical studies to preserve β‐cell function and maintain lower HbA1c for up to 2 years, compared to placebo, in new onset T1D,^72^ and is effective in children as young as 5 years of age.^73^ Abatacept is a fusion antibody that blocks T‐cell activation, that has been shown to delay decline of C‐peptide in individuals with Stage 3 T1D, when administered monthly for 2 years,^74^, ^75^ but showed no delay in disease progression in individuals at Stage 1 T1D.^76^ Golimumab, an antibody specific for TNFα, and the Janus kinase (JAK) inhibitor baricitinib have also been shown to preserve β‐cell function in RCTs on individuals with new‐onset T1D, compared to placebo.^77^, ^78^ A number of other immunotherapies have been tested or are currently under investigation in RCTs, with variable outcomes to date.^79^ Outside immunotherapies, other drugs, such as the calcium‐channel blocker Verapamil, have shown significant preservation of residual β‐cell function in Stage 3 T1D, both in young adults^80^ and children,^81^ and are now under investigation in Stage 1 and Stage 2 T1D.

## 5 REDUCING THE COST BURDEN OF TYPE 1 DIABETES THROUGH ISLET AUTOANTIBODY SCREENING

Although calculating the financial burden of T1D in Europe is subject to diverse modelling, several studies evaluating the combined direct and indirect costs have derived remarkably similar estimates of between €6000–7000 per person annually over the last 5 years,^82^, ^83^, ^84^ with projections that this could rise to €12 057 by 2040.^84^ Based on the reported incidence rates, this suggests an annual cost burden of approximately €17 billion across Europe, rising annually and excluding indirect costs such as lost workforce participation due to absenteeism caused by diabetes related illness. Workplace productivity is a significant additional cost, with early exit from the workforce being 62% more likely for a person with T1D, compared to a person without diabetes.^85^ The costs associated with diabetes‐related absences from the workplace are not well researched, and have been variably estimated to be from one‐third^83^ to three‐fold those of diabetes‐related healthcare costs.^85^

**FIGURE 2:**
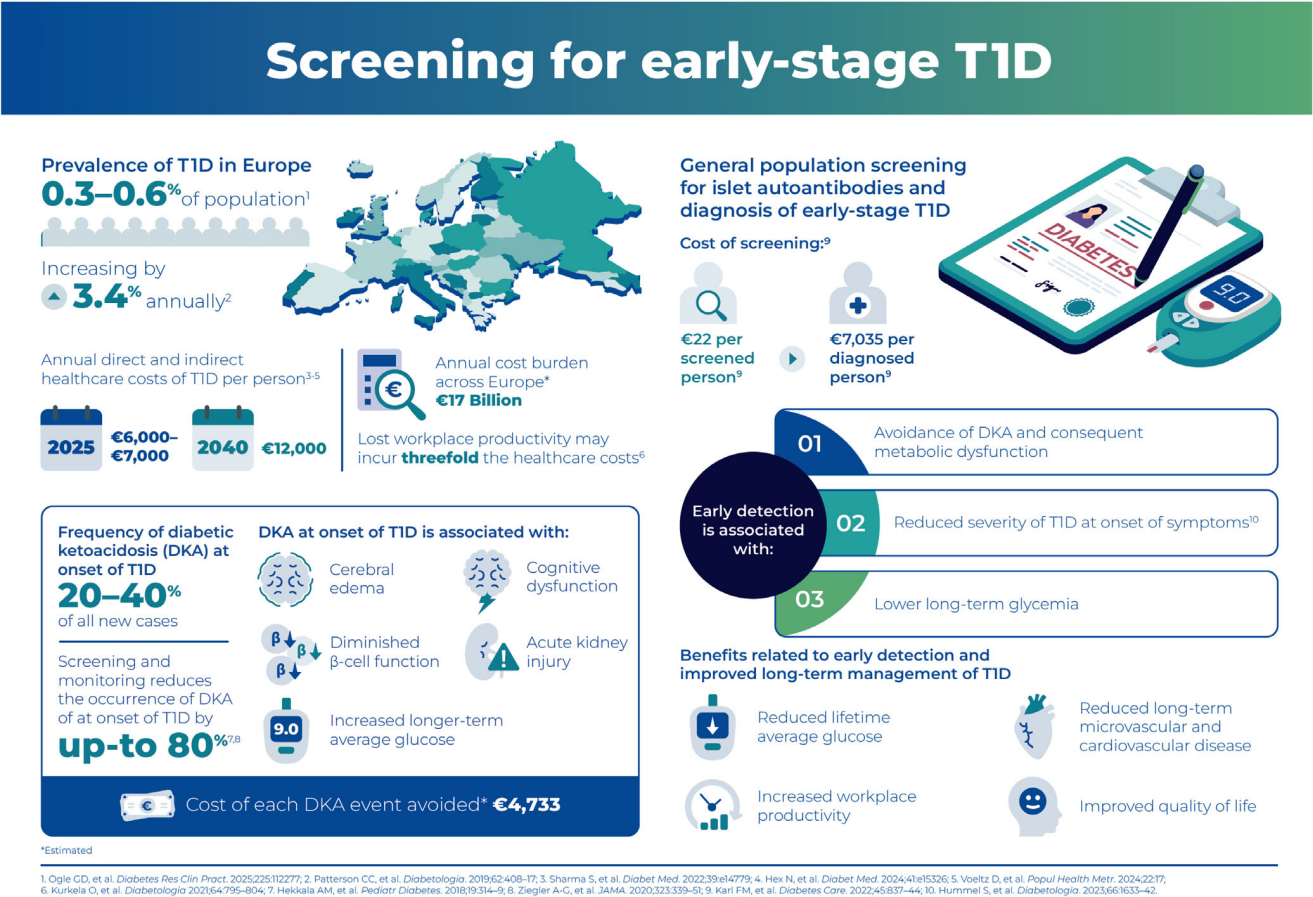
Screening for early‐stage T1D.

**FIGURE 3:**
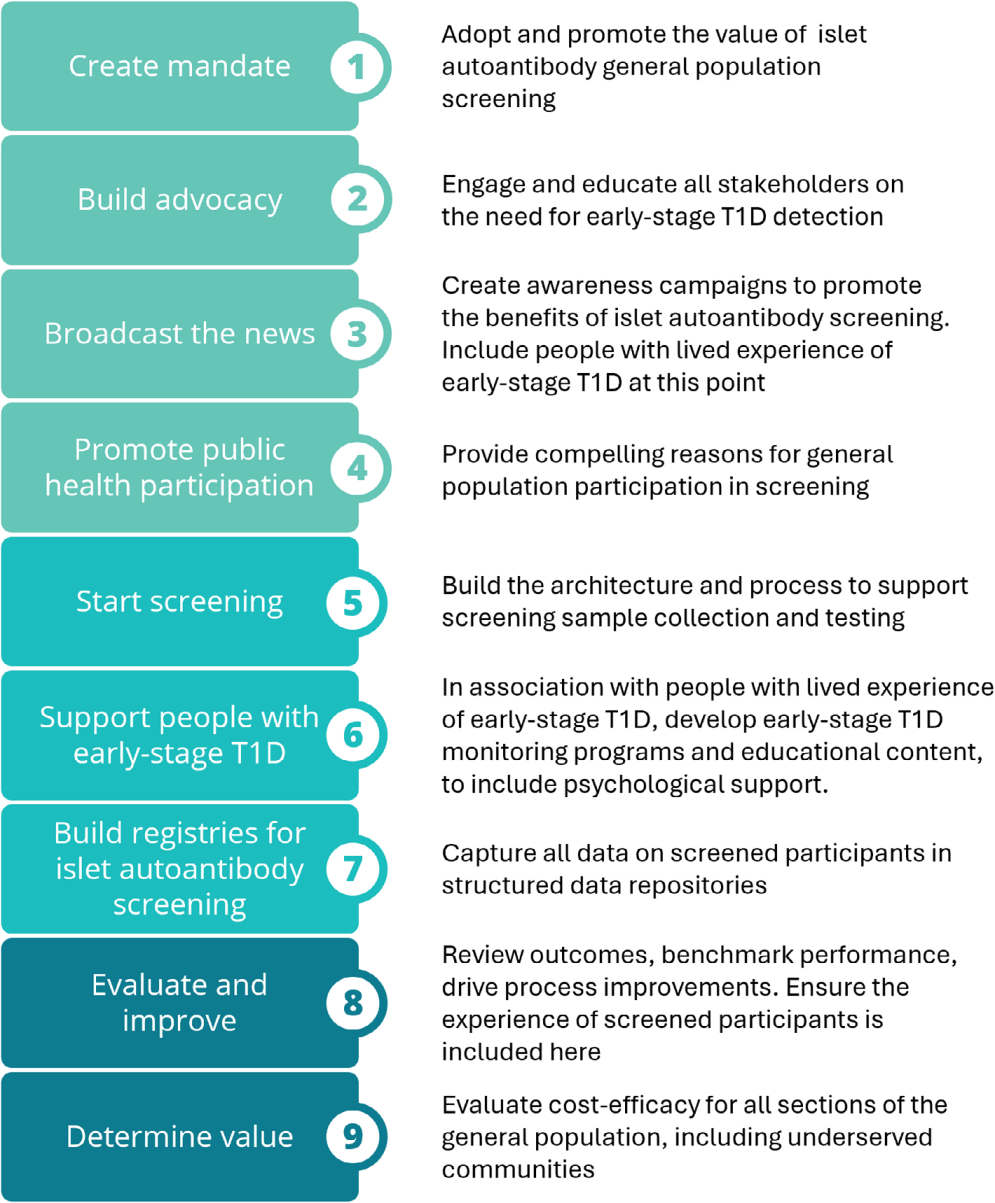
A roadmap for implementation of islet autoantibody general population screening. T1D, type 1 diabetes

The key elements of the value proposition for early detection of T1D are summarized in Figure 2 and the overall process steps for implementation of islet autoantibody general population screening are outlined in Figure 3. When considering the cost‐efficacy of general population screening programs, a range of inputs and outputs must be considered. These include the screening pathway and the costs to maximize participation, the potential consequences of minimizing false‐positive and false‐negative test results, the costs of treating and optimizing outcomes for individuals with early‐stage T1D, as well as non‐health benefits and harms. Many of the significant benefits of early diagnosis of T1D through islet autoantibody screening are a consequence of participation in monitoring studies, and these must be set up and available from the start of screening, with associated education for all healthcare professionals (HCPs) who participate in screening and monitoring of early‐stage T1D.

The outcomes of early detection of T1D have broad societal implications, with short and long‐term reductions in healthcare costs and increased workforce participation and productivity.^86^, ^87^ Early awareness of their autoantibody status has significant benefits for the person who has screened positive. These include the opportunity to participate in education for symptom awareness and monitoring of disease progression (see above), such that the incidence of DKA is significantly reduced at development of symptomatic hyperglycemia^19^ which also reduces the economic impact on healthcare services. Another important outcome of early awareness and management of glycemia is the potential improvement in long‐term glycemic outcomes for individuals with T1D following the start of insulin therapy, with median HbA1c levels 5.0 mmol/mol (0.5%) lower after 5 years of follow up, compared to individuals not participating in monitoring.^88^ These reductions in long‐term glycemia lower the risks for microvascular and macrovascular complications of T1D, which together comprise 20% of the total costs of treating T1D.^83^

An important consideration when making cost‐efficacy determinations for general population islet autoantibody screening is the overall cost for detection of each case of early‐stage T1D. With estimated detection rates of 0.3–0.6% of all screened individuals,^19^, ^86^ this means that >99% of all general population screening tests will be negative. These tests must still be accommodated in the overall cost–benefit analysis for the screening program.

Health economic modelling based on cohorts with genetic risk scores likely do not provide strong evidence for the cost‐efficacy of islet autoantibody general population screening,^89^ which relies on limited analysis from general population cohorts. One study from the ethnically diverse ASK general population cohort in Colorado has assessed that islet autoantibody screening is cost‐effective for US payers if it lowers the rate of DKA in the screened population by 20% (e.g., from 35% to 28% of new‐onset T1D cases) and reduces HbA1c by 0.1% over a lifetime, with associated avoidance of microvascular and macrovascular complications and costs.^86^ In reality, reported rates of DKA for individuals who participate in screening with follow‐up monitoring, compared to DKA rates in comparable unscreened community dwellers, are at least 44% lower,^21^ and 5‐year reductions in HbA1c are 0.5%.^88^ From a European perspective, the Fr1da general population screening study in Bavaria has been evaluated as cost effective,^87^ based on a €22 screening cost per child screened and a €7035 cost per child diagnosed with early‐stage T1D. Minimizing screening laboratory costs was important in both the ASK and Fr1da cohorts. The ASK cohort screening was largely performed by radiobinding microassay on serum samples,^86^, ^90^ whereas the Fr1da population screening assay was based on the 3‐Screen islet‐cell autoantibody ELISA using serum samples.^87^ In the ASK cohort the cost of screening was the key driver of cost‐efficacy, whereas for the Fr1da study cohort the main driver was the HCP provider costs for managing the screening activity. In neither situation were indirect costs for increased lifetime workplace productivity included in the analysis. There is insufficient data to model the cost‐impact of disease modifying treatment with teplizumab at this time.

## 6 THE LIVED EXPERIENCE OF ISLET AUTOANTIBODY SCREENING FOR EARLY‐STAGE TYPE 1 DIABETES

A 2024 study surveyed the knowledge and attitudes of 510 people affected by T1D, across three groups, and their experience of islet autoantibody screening.^91^ These included parents living with T1D and a child without T1D, caregivers of a child with T1D and at least one other child not with T1D, and first‐degree relatives (FDRs) of a person with T1D. Most parents with T1D and FDRs reported little or no knowledge about islet autoantibodies (51% and 76%, respectively), and 12% or less considered themselves very or extremely knowledgeable. Caregivers with a child with T1D were more likely to be very or extremely knowledgeable about islet autoantibodies (24%), and a further 48% of this group indicated they were somewhat knowledgeable. However, more than 70% of all participants expressed a positive or very positive general attitude to autoantibody screening. Regarding the knowledge gap, a smaller survey of 38 parents of children invited to participate in the ELSA general population T1D autoantibody screening program in the UK revealed a lack of understanding of the type of diabetes associated with islet autoantibody screening.^92^

Only a minority of survey respondents (29%) had actively participated in screening. Parents with T1D and caregivers reported positive experiences of the screening sample collection method (68% and 73% respectively), the majority of which were a blood draw in a provider's office or a fingerstick capillary test at home. FDRs reported neutral (40%) or positive (53%) experiences, and negative experiences were reported in only 8% or less of all cases. Tellingly, 93% or more people who had taken part in screening would do so again.

Among people who had not participated in screening, caregivers and FDRs were uncertain about future participation, with only 29% and 16% indicating they planned to undertake screening, and 30% and 40% indicating they were unlikely to participate. Parents with T1D were more likely to have their child without diabetes take part in screening (49% probably or definitely), with only a small minority (13%) indicating they probably or definitely would not participate. These outcomes did reveal discordance between the positive general attitude towards autoantibody screening and the more personal intentions of parents with T1D, caregivers, and FDRs.

Across the survey, the low rate of participation in screening (71%) was of interest. Caregivers were most likely to have their children without T1D screened (46%), whereas only 16% of parents with T1D had their own biological children screened. This may reflect the relative difference between these groups regarding knowledgeability of islet autoantibodies, which was considerably higher for caregivers (24% vs. 12%). The low rate of active screening participation among FDRs (19%) may be related to low knowledge of islet autoantibodies in this group (76%), or perceptions that adult onset T1D was less likely, as expressed by survey participants. However, since at least half of new T1D diagnoses are in adults,^93^ this also reveals a knowledge gap and a need for additional education.

An important outcome of the survey is the positive experience of people who had participated in screening, including the blood sampling method. The typical outcome of screening for this group was a determination of no autoantibodies, which may have biased responses. However, this is also the anticipated outcome of large‐scale general population screening, and the attitudes of people who have undertaken screening should be leveraged as part of awareness campaigns in support of general population screening.

## 7 ACCEPTABILITY AND FEASIBILITY OF SCREENING

Given the necessary involvement of HCPs, an international online survey of attitudes among diabetes aware HCPs revealed some interesting observations.^94^ Across 431 respondents, 82% strongly supported islet autoantibody screening in children and adolescents for close relatives of people with T1D, but only 26% were strongly supportive for general population screening, with a further 36% undecided. Significantly, respondents' support for general population screening was much stronger if they felt such programs were available to them. These proportions reflected the international viewpoint and those of the 202 European respondents. No information was available on the levels of education or awareness of the benefits of general population screening among respondents.

One of the Wilson and Jungner principles of general population screening (Table 1) is that the test should be acceptable to the screened population, that is, it has a low burden for participation. A report on a feasibility and acceptability pilot for the UNISCREEN general population screening for T1D and celiac disease in Italy^95^ reported overall satisfaction with the purpose of screening and the process, with 90% of participants preferring capillary fingerstick sampling as simple and practical, and preferable to venous sampling. Prior to screening, a proportion of participating adults and parents of children reported anxiety about the possibility of autoantibodies being detected. For most people, this anxiety and worry decreases with time but some individuals may be particularly vulnerable to prolonged anxiety or depression.^96^

Parents of children participating in the ELSA general population screening project^92^ expressed positive attitudes to screening that were associated with clear communication and education on the purpose and outcomes of screening for T1D, including a better understanding of the differences between T1D and T2D. Prior participation in other public health programs also increased motivation. Fingerstick capillary blood sampling using at‐home kits, with return of dried blood spot (DBS) test cards, has also been shown to be acceptable to participants in autoantibody screening.^97^

## 8 THE BENEFITS OF SCREENING AND PARTICIPATING IN MONITORING PROGRAMS IN EARLY‐STAGE T1D

Screening not only improves clinical outcomes but may also reshape the diagnostic experience, allowing families to enter the T1D journey more informed, emotionally supported, and connected to care. For people who screen positive for islet autoantibodies as part of general population screening, participation in organized monitoring programs has significant value,^16^ since there is no obvious mechanism to predict when a person with early‐stage T1D (Stage 1 or Stage 2) may progress to Stage 3 T1D. For children and adolescents, the persistent presence of multiple autoantibodies is associated with higher rate of progression to Stage 3 T1D.^98^ Monitoring the type of positive autoantibody is also of importance – since as children age, relative risks for progression with each autoantibody type will change,^99^, ^100^, ^101^ with some evidence that this is also true for adults.^99^, ^102^ The limited data available suggest that the rate of progression to Stage 3 T1D in adults who screen positive for early‐stage T1D (Stage 1 or Stage 2) is slower than in children.^103^, ^104^

Across screening and monitoring studies, one of the highest value outcomes of participation for individuals who have screened positive for early‐stage T1D (Stage 1 or Stage 2) is the dramatic reduction in the incidence of DKA following progression to Stage 3 T1D. For young children aged <2 years at onset of Stage 3 T1D, participation in The Environmental Determinants of Diabetes in the Young (TEDDY) monitoring study resulted in a significantly lower rate of DKA (15%) compared to incidence reported in national diabetes registries in countries where TEDDY participation was available (Sweden, 40%; Finland, 45%; Germany, 54%).^33^ Similar outcomes were seen in children <5 years old. In Finland, children enrolled in a prospective monitoring study following newborn screening for human leukocyte antigen (HLA)‐associated risk for T1D had a 5.0% incidence of DKA at onset of clinical T1D during a 15‐year follow‐up period, compared to 23.4% of children screened positive for HLA‐associated risk of T1D but who did not participate in monitoring.^105^ In the Fr1da study, a general population screening study in Bavaria, Germany,^19^ among children screened autoantibody positive for early‐stage T1D (Stage 1 or Stage 2), only 3.2% were diagnosed with DKA on progression to Stage 3 T1D, compared with reported DKA prevalence rates of 20.8–35.6% nationally for Germany (Table 2).

A component of participation in monitoring programs and studies is the heightened awareness and education that is provided regarding the symptoms and signs of DKA. Families of children who screened positive for early‐stage T1D (Stage 1 or Stage 2) in the Fr1da study,^19^ were invited to participate in an educational program on metabolic stages of T1D, where they received training in urine and blood glucose monitoring, information on normal and abnormal blood glucose levels, and the symptoms of hyperglycemia and DKA.^106^ This was accompanied by a guidebook specifically designed for children with early‐stage T1D and assigned a local contact from the diabetes center to whom they could ask questions at any time. The children and families who did not participate in education and metabolic staging had an increased rate of DKA and significantly increased length of hospital stay on progressing to Stage 3 T1D.^20^, ^105^ Within TrialNet, follow‐up and monitoring of antibody‐positive relatives, with Stage 1 and Stage 2 T1D, at a single centre, with frequent contacts over the years resulted in complete prevention of DKA in those who progressed to Stage 3 T1D.^107^

## 9 DIABETES REGISTRIES MUST BE CREATED OR ADAPTED TO MANAGE DATA FROM EARLY‐STAGE T1D SCREENING PROGRAMS

In a European context, a critical component of effective general population screening for early‐stage T1D is the collection of data on all screened individuals, whether the results of their screen is that no islet autoantibodies were detected, or if single or multiple autoantibodies were detected. The need is for a structured data capture format (hereafter referred to as a registry) that can be standardized and implemented across separate European regions. The primary purpose is to allow screened individuals to be identified, with outcomes and actions associated with the screening result, as well as to enable follow‐up as needed. This will include risk assessment for people diagnosed with Stage 1 or Stage 2 T1D, initially based on accumulated data from ongoing general population registry studies, such as Fr1da and ASK, but also from studies centered on genetically risk scored individuals. An important outcome of the general population early‐stage T1D registry data is to evolve risk prediction for new‐onset Stage 3 T1D using general population data and outcomes. Within this context, early‐stage T1D registry data can be used to benchmark the efficacy of disease monitoring programs and disease modifying therapies, where available and applied, and to build models of care and cost‐efficacy.

The European Diabetes Forum (EUDF) has made the case for starting or evolving diabetes registries covering people with clinical T1D or T2D,^108^, ^109^ with the goal of pan‐European application, and the same principles and practice are applicable to registries containing pseudonymous data on people who have participated in screening for early‐stage T1D, including those who have screened positive or negative for multiple islet autoantibodies. Importantly, issues centered on compliance with General Data Protection Regulation (GDPR) and the 2025 European Health Data Space (EHDS) regulations on access, sharing and use of electronic health data across European Union member states are addressed in this context.

To date, European registries that specifically incorporate the outcomes of general population islet autoantibody screening include the Fr1da study,^19^ the United Kingdom Islet Autoantibody (UKIAb) registry (www.ukiab.org) and the EDENT1FI pre‐T1D‐registry (www.pre-t1d-registry.eu).^110^

## 10 LEVERAGING THE IMPACT OF PUBLIC AWARENESS CAMPAIGNS IN DKA PREVENTION AT DIAGNOSIS OF T1D

To optimize the benefits of a public health screening program, it is critical to encourage participation. Research among individuals with experience of T1D in their family indicates that lack of understanding of the purpose of islet autoantibody screening was a barrier to their own participation in screening.^91^ The initial outcomes from the Italian D1Ce autoantibody screening pilot study^111^ have shown that early awareness and engagement among family pediatricians and other stakeholders resulted in a significant 16% reduction in DKA admissions for new‐onset T1D in participating regions, compared to non‐participating regions, even though the screening process had not started.^112^ The use of targeted awareness campaigns to increase general population participation in screening programs can deliver further gains.

A systematic review of studies across European territories reporting changes in incidence of DKA at diagnosis of T1D as a result of diabetes awareness campaigns^113^ found that implementing public awareness campaigns could result in reductions in DKA at onset of T1D by up to 65.5%. Effective campaigns were associated with a well‐defined focus on parents, school teachers and HCPs regarding symptoms of T1D or DKA (polyuria, polydipsia and enuresis) in children.^114^, ^115^, ^116^, ^117^ Prevention and awareness campaigns should also be carried out in regional rather than national areas, and should last at least 2 years, renewed every 5 years.^115^, ^116^, ^118^ Multiple awareness tools should be provided, including posters and flyers, targeted educational meetings and events, hands on demonstration of urine and capillary blood testing, posts on social media and radio, and free call‐lines to diabetes centers.^114^, ^115^, ^116^, ^119^ Campaigns should also be actively monitored, to ensure that the target population has seen or heard the awareness media, and to evaluate the effectiveness of the campaign.^116^ The most successful campaigns mounted in Europe have reported reductions of 40–60% in the frequency of DKA at diagnosis of T1D,^117^, ^119^, ^120^ as well as reduced severity of DKA,^121^, ^122^ and fewer neurological complications.^118^

## 11 EDUCATION AND AWARENESS FOR CLINICIANS AND ALLIED HEALTHCARE PROFESSIONALS

As much as awareness and understanding of the needs for islet autoantibody screening are important within the participating general population, the needs for education among HCPs are equally essential. HCPs are time poor, particularly those in primary care, and they must fully understand the process if it is to deliver the proposed benefits. The availability of disease modifying therapies for early‐stage T1D is an important part of this step‐change in HCP awareness and education. Even within healthcare teams with experience of managing people with diabetes, awareness of the principles and practice of managing early‐stage T1D is not established. A UK healthcare professional survey covering 66% of pediatric diabetes units (PDUs) reported that 69% of PDUs that reported managing children and adolescents with early‐stage T1D were district general hospitals, compared to 31% of PDUs that were tertiary hospitals.^123^ Notably, the survey revealed significant heterogeneity of management strategies for children with confirmed islet autoantibodies, reinforcing the need for consistent education about early‐stage T1D across the HCPs in these services. For example, only 24% of respondents indicated that they provided education for islet autoantibody positive children and carers, and only 13% recommended referral to a research study.^123^ The key concepts around monitoring and managing asymptomatic early‐stage T1D must become embedded for HCPs, particularly those in primary care on whom the responsibility of dealing with general population screening is likely to fall. It is imperative that this group understand and accept that autoantibody screening and identification of early‐stage T1D, with subsequent participation in education and monitoring programs,^16^ improves glycemia and preserves β‐cell function at the onset of symptoms, with significantly reduced rates of DKA, compared to age‐matched children diagnosed with T1D without prior screening.^19^, ^20^ Following the start of active screening, a greater reduction in overall incidence of DKA can be observed as an indirect effect associated with the campaign, as recently shown in Italy.^112^ This suggests that improved awareness of the signs and symptoms of new‐onset T1D can reduce the risk of DKA, independent of confirmation of early‐stage T1D in screened individuals. Consensus guidance for the management of children, young people, and adults with early‐stage T1D is available,^16^ and defined behaviors for HCPs participating in pre‐screening and post‐screening activities have been developed.^124^ These, and other resources developed for general population screening for early‐stage T1D, must underpin the activities of all participating HCPs.

## 12 EDUCATION AND COMMUNICATION FOR GENERAL POPULATION PARTICIPANTS

The immediate and long‐term benefits of general population screening are evident from the outcomes of participation in education and monitoring, as discussed earlier. Effective communication around these benefits during the pre‐screening period can optimize participation among the general population.^91^, ^125^ Similarly, the low risks of autoantibody detection must be made clear. Engagement with general population screening is associated with proactive communication and education on the purpose and outcomes of screening for T1D.^92^ A significant unmet need in understanding the role of education and communication in general population screening is the objective and qualitative perceptions of the communities of people living with T1D and their position regarding public health screening. This is an essential component of understanding both the challenges and benefits of early‐stage T1D screening from the perspective of this key advocate community.

The need for education in the immediate post‐screening period is significant. For someone with a positive screen, this should include as much information as possible on the types of islet autoantibodies for which they have screened positive, and what this means. Communicating the results of a negative result must include awareness of the need for routine autoantibody screening in the future, and that a negative screen is not a guarantee that the individual will never develop T1D.

## 13 PSYCHOLOGICAL SUPPORT ASSOCIATED WITH PARTICIPATION IN ISLET AUTOANTIBODY SCREENING

For individuals who have screened positive for islet autoantibodies, the psychological and glycemic benefits of participating in monitoring programs or early‐stage T1D clinical studies have been discussed above and are covered in thorough recommendations.^16^ In fact, the primary goal of providing information and education following a positive screen is to drive engagement with such monitoring programs.^16^ Parents of children participating in a general population islet autoantibody screening project were more likely to engage with screening if psychosocial support was made available should their child have early‐stage T1D.^92^ Without such support, individuals and their families can experience anxiety and a sense of helplessness following detection of islet autoantibodies, living in a state of ‘suspended uncertainty’—aware of the elevated risk yet, in most cases, unable to prevent the onset of T1D. Many may develop anticipatory anxiety related to the unpredictability of the disease,^126^ which can affect their daily functioning, strain family relationships, and lead to excessive monitoring of health behaviors.^96^, ^126^, ^127^

Accepting and adapting to a diagnosis of early stage T1D can positively impact daily health and quality of life. Preliminary research indicates that sharing experience and reciprocating support among peers also has high value for parents and carers with children who participate in islet autoantibody screening.^128^ In general population screening programs, psychosocial support may need to be further emphasized among parents with lower indices of educational attainment and from racial or ethnic minority backgrounds.^126^

## 14 ONGOING STUDIES FOR GENERAL POPULATION SCREENING FOR ISLET AUTOANTIBODIES FOR T1D

A number of screening programs are underway in Europe, as detailed in Table 3. Many have been focused on the detection of islet autoantibodies in children with a familial history of developing T1D. Others, like the Global Platform for the Prevention of Autoimmune Diabetes (GPPAD) and the Finnish Diabetes Prediction and Prevention Study (DIPP), take a few drops of blood from the heel or hand of newborns within a few days of birth and test for genetic markers of risk for T1D.^129^, ^130^ At‐risk newborns are then invited to participate in intervention trials aimed at preventing progression to T1D, including testing for islet autoantibodies. It is important to note that such genetic screening does not itself detect early‐stage T1D, which is only possible using islet autoantibody screening. Newborn screening mandates are common for many rare genetic diseases.^131^ However, most individuals who develop T1D do not have any genetic risk markers, and only islet autoantibody screening can detect early‐stage T1D. In this context, islet autoantibody detection is unrealistic in newborns, provided antibodies at birth are primarily from maternal transmission.^132^ The number of studies that are centered on recruitment from the general population is growing significantly, including several focusing on adults, such as UNISCREEN.^133^ The outcomes from these general population studies will greatly inform the principles and practice of general population screening, and initial data from the D1Ce screening pilot in Italy has shown reductions in DKA within the screened populations.^112^

**TABLE 3:** Summary of European based clinical studies on detection of islet autoantibodies to diagnose early‐stage T1D.

Study	Location	Scope and participation	Insights to date	Stakeholder groups
Fr1da	Initiated in Bavaria, Germany, extended to Saxony, Lower‐Saxony, and Hamburg	Screening for multiple islet autoantibodies in children 2–5 years initially. Currently available to children 2–10 years old.	Screening for early‐stage T1D is feasible. Reduced DKA at diagnosis of Stage 3 T1D (3.2% vs. >20%). 18% fewer days in hospital. 60% reduction in symptom days before Stage 3 diagnosis. 71% reduction in number of children with weight loss prior to diagnosis of Stage 3 T1D.	Professional Association of Primary Care Pediatricians, pediatric diabetes care centers and the Ministry of Health of Bavaria Part of EDENT1FI
Diabetes Prediction and Prevention Study (DIPP)	Finland	Screening for newborn infants with close family relatives with T1D. Started 1994.	In at‐risk children, risk of developing islet autoantibodies is decreased by consumption of cruciferous vegetables and berries.	
DiaUnion/TRIAD Study	Sweden & Denmark	Early diagnosis of T1D, celiac disease and autoimmune thyroid disease in the general population. Includes screening of: (a) randomly selected children in Sweden, and (b) first‐degree relatives people with T1D from the Danish Registry for Children and Adolescent with Diabetes.	2.6% of 2271 children randomly screened were islet autoantibody positive. Autoantibodies more frequent in 6–9 year old group. Stage 3 T1D detected in 3 participants, all 6–9 yrs old.	Lund University (Sweden) and Steno Diabetes Centre (Denmark). Part of EDENT1FI
European action for the Diagnosis of Early Non‐clinical Type 1 diabetes For disease Interception (EDENT1FI)	Pan‐European	General population islet autoantibody screening in 200 000 children/adolescents aged 1–17 years. Centers in Czech Republic, Denmark, Germany, Italy, Poland, Portugal, Sweden and UK. Started November 2023 and planned to conclude in October 2028.	Still recruiting.	European Commission Innovative Medicines Initiative Joint Undertaking (IMI‐JU) The Hemsley Charitable Trust Breakthrough T1D UKRI Guarantee Fund
RADAR1	Portugal	Screening for children and young people, aged 3 to 17 years, with first degree relatives with T1D.	Still recruiting.	Associação Protectora dos Diabéticos de Portugal (APDP)
EarLy Surveillance for Autoimmune diabetes Study (ELSA)	United Kingdom	General population islet autoantibody screening Recruiting since Nov 2022. Aims to screen 20 000 children aged 3–13 years. DBS collection at home, hospital or community settings.	Still recruiting.	Part of EDENT1FI
Type 1 Diabetes Risk in Adults (T1DRA)	United Kingdom	General population islet autoantibody screening. Aims to screen 20 000 adults aged 18 to 70 years. DBS collection at home, hospital or community settings.	Still recruiting.	The Hemsley Charitable Trust. Diabetes UK.
UK Islet Autoantibody Registry (UKIAb)	United Kingdom	Registry of children, young people and adults with early‐stage T1D detected by IAb in research studies or clinical care. Offers confirmatory autoantibody testing to all children and adults in a reference laboratory.	Ongoing recruitment and management of IAb positive individuals; resource to offer prevention trials. Substudies include: qualitative study in adults, parents, children and young people; data linkage on healthcare utilization; progression modelling.	University of Oxford, Centre for Human Genetics. Diabetes UK.
βetty study	Czechia	Pilot general population screening program aimed at the early detection of T1D in children aged 2–18 years. Founded 2023.		EU Horizon grant. Part of EDENT1FI.
BABYDIAB/BABYDIET	Germany	Screening for newborn infants with first degree relatives with T1D.	By age 2 years, 11% of children had at least one autoantibody and 3.5% had multiple autoantibodies. Children with multiple autoantibodies by age 2 had a 50% risk of developing T1D by age 5.	
INNODIA Natural History Study	Pan‐European	Multicenter study involving 18 diabetes centers across Europe, with 47 active clinical sites overall. Adult and pediatric recruitment from Nov 2016‐Nov 2021.	Overall prevalence of DKA at diagnosis of T1D is 36%. 23% prevalence of DKA at diagnosis of T1D in adults. 44% prevalence of DKA at diagnosis of T1D in children aged 10–17 years.	European Commission Innovative Medicines Initiative Joint Undertaking (IMI‐JU). The Hemsley Charitable Trust. Breakthrough T1D. European Federation of Pharmaceutical Industries and Associations (EFPIA).
Global Platform for the Prevention of Autoimmune Diabetes (GPPAD)	Germany, UK, Sweden, Belgium, Poland	Screening of newborns for genetic risk of T1D and enrolling eligible infants into prevention trials.	POInT trial set up to determine if oral insulin can induce immune tolerance and reduce the development of islet autoantibodies. Enrolled 1050 infants with genetic risk for T1D.	The Hemsley Charitable Trust.
UNISCREEN (Universal screening for early detection of chronic autoimmune, metabolic and cardiovascular diseases in the general population using capillary blood)	Italy	Population‐wide pilot screening initiative assessing the feasibility and acceptability of capillary blood testing for early detection of chronic diseases, including early stage T1D and celiac disease.	Among 1535 screened individuals, 90% of capillary blood tests were successful. Hypertension and dysglycemia detected in 60% and 21% of participants.	Fondazione Italiana Diabete (FID).
D1Ce Screen (propaedeutic of National Italian program)	Italy	Screening of 5500 children for T1D and celiac disease at ages of 2–3, 6–7, 10–11 years in four regions (Lombardy, Marche, Campania, Sardinia).	Preliminary observations show stakeholder engagement and awareness activities reduced DKA for new‐onset T1D by approx.16%. Screening activities reduced DKA for new‐onset DKA by approx. 22%.	Ministry of Health/Istituto Superiore di Sanità; Professional Association of Family Paediatricians; Fondazione Italiana Diabete; Italian Society of Paediatric Endocrinology and Diabetes; Part of EDENT1FI
SCREEND1A	Spain – Pais Vasco	Pilot program in General population screening for four islet autoantibodies in children aged 3 to 17 years old.	Still recruiting.	Pais Vasco Government Health research Division.

Abbreviations: DBS, dried blood spot; DKA, diabetic ketoacidosis; EU, European Union; T1D, type 1 diabetes; UK, United Kingdom.

## 15 ENSURING ACCESS AND ACCESSIBILITY

The benefits of early detection of T1D and the availability of disease modifying therapies require that access to islet autoantibody screening is available to all individuals across all socioeconomic sections of the population, and in all urban, rural or remote locations.^134^ Underserved communities, including those from racial and ethnic minority backgrounds, or with higher indices of socioeconomic deprivation, are clearly shown to bear a disproportionate burden of diabetes.^135^, ^136^ Rates of DKA at first presentation of new‐onset T1D are increased in rural and deprived populations,^32^, ^137^ with one assessment assigning a 42% increase in rates of DKA at diagnosis of T1D based on rural location.^27^

In ensuring access, it is likely that several screening options may be necessary. Participation via mainstream public health initiatives, such as that mandated by the Italian Parliament and managed by the Italian Health Ministry through the National Health System (SSN), is an established solution.^138^ The expectation is that participants will undertake screening by attendance at a scheduled event, such as a well‐child clinic or vaccination. For children, adults, and families unable to participate in this way, referral to an ongoing early‐detection study may be possible in their region (Table 3), with the option of a visit from a healthcare team member or an at‐home self‐test using a capillary blood sample,^26^ or other acceptable method.^95^ In all eventualities, access to any testing service will require that public awareness campaigns are effective and that HCPs are themselves educated and aware of the need for islet autoantibody screening and able to answer questions and direct the participant to the most accessible screening choice.

## 16 UNMET NEEDS FOR FURTHER RESEARCH AND POLICY

A considerable unmet need is for clinical studies to detect early‐stage T1D in general populations not selected for genetic or higher‐risk profiles for T1D, and for adult participation in such studies. The majority of clinical data and research outcomes to date have been derived from prevention studies in children and adolescents with close relatives with T1D, or with known HLA class II haplotypes associated with T1D. The Fr1da and ASK studies are notable exceptions in this regard. Similarly, there is a need for wider inclusion of diverse groups from non‐Caucasian backgrounds in screening and prevention studies. The treatment responses of Hispanic, Black, Asian, and other groups with non‐European ancestry will also require further investigation. From a policy perspective, there is a significant need for diabetes registries that can incorporate data from early‐stage T1D screening programs to facilitate benchmarking and cost‐impact measures for evaluation of disease‐modifying therapies and disease monitoring programs. The impact of using disease‐modifying therapies to delay or prevent progression of early‐stage T1D to new‐onset Stage 3 T1D is a significant unmet need that has important ramifications for the health and wellbeing of people who screen positive for islet autoantibodies, as well as for the long‐term costs and cost‐effectiveness of general population screening.

**Recommendations**

**General Principles**

1. Islet autoantibody screening for early‐stage T1D should promote equity of access for all individuals, independent of socioeconomic status, ethnicity, or regional location.
2. Healthcare policymakers should further investigate and engage with the principles and practice of general population screening for early‐stage T1D. Policy initiatives should be embedded in healthcare systems within individual European countries and as part of pan‐European public health programs.
3. The value of early‐stage T1D detection at a general population level should be part of education for all healthcare professionals

**Infrastructure**

4 Programs for general population screening for early‐stage T1D must be supported by an infrastructure for initial screening tests, follow‐up confirmation and monitoring for those with detectable islet autoantibodies, which optimize the benefits of detection of early‐stage T1D.
5 Diabetes registries must be adapted or created that capture all of the data generated by general population screening, in line with European and/or regional regulatory requirements. These data registries/repositories should clarify the outcomes for all participant communities and demographics, and be structured for optimal data sharing across national and international boundaries, if and when an appropriate written consent from the individual and/or legal guardian is obtained
6 Awareness campaigns should clarify and explain the benefits of early detection of islet autoantibodies, prior to initiating general population screening programs. These should reflect the lived experience of participants in screening programs for early‐stage T1D and be adapted to national and regional differences in healthcare service infrastructure, and recognize the important roles of primary and secondary care.

**Participation**

7 All participants in general population screening for early‐stage T1D should be provided with clear information about identifying the symptoms of T1D.
8 For individuals and families with confirmed early‐stage T1D, education and support must be provided that clarifies the meaning of this diagnosis and what to expect in the short and long‐term. This should be developed in association with people with lived experience of screening positive for islet autoantibodies. This must also recognize the potential for anxiety and distress, with integrated psychosocial support.
9 Individuals and families with confirmed early‐stage T1D must receive information on potential interventions for delaying or modifying the progress of early‐stage T1D to clinically overt Stage 3 T1D, available either in routine care or as part of ongoing clinical trials.

**Evaluation**

10 Once initiated, early‐stage T1D screening programs should collect person‐reported outcomes (PROMs) on the experience of participating in screening in order to understand the benefits and barriers to participation independently of the outcomes of screening.
11 The outcomes of general population screening for early‐stage T1D should be reviewed annually to establish the prevalence and impact of early‐stage T1D, benchmark healthcare service performance in monitoring and mitigating adverse consequences of symptomatic T1D, and to drive service improvements in detecting and managing T1D. Formal reports to national health authorities are prudent.
12 The costs and benefits of screening for early‐stage T1D must be evaluated at local, national and pan‐European levels, with clear reporting for each stakeholder community.

## 17 CONCLUSIONS

WHO principles for public health screening for chronic conditions are close to being met for T1D and the availability of disease modifying therapies for early‐stage T1D, prior to the development of symptomatic clinical disease, makes general population screening for islet autoantibodies a compelling and ethically bounded proposition. Evidence shows that early detection of preclinical T1D and subsequent management of people with detectable islet autoantibodies has a significant impact on the occurrence of acute diabetes events such as DKA once clinical symptoms develop, and also has long‐term benefits for reduced glycemia and lessened risks for chronic complications of T1D. Across Europe, regional islet autoantibody screening programs are underway, which collectively help us to identify a roadmap for wider implementation (Figure 3). The key components of this roadmap include significant investment in education and awareness, both for HCPs and for the general public, along with new diabetes registries that can support outcomes research on new therapies and intervention strategies. Incorporating the lived experience of people who have participated in early‐stage T1D screening is an important part of this roadmap. This is the moment for wider application of general population islet autoantibody screening and to create national and international goals for reducing the human and economic burden of T1D in Europe.

## AUTHOR CONTRIBUTIONS

The authors contributed edits to the interpretation of key concepts and supporting research over serial drafts of the final manuscript. SH and TB are the guarantors of this work and take responsibility for the integrity of the analysis. All authors have reviewed, helped to revise, and have approved the final version of this manuscript.

## FUNDING INFORMATION

This initiative was led by the International Diabetes Federation Europe (IDF‐Eu) and supported by a grant from Sanofi, who had no involvement in the development of the content or the recommendations.

## CONFLICT OF INTEREST STATEMENT

Sufyan Hussain is an IDF Europe Board Member; he has served on the advisory board for Tandem, Dexcom, Medtronic, Sanofi, Roche, Vertex; undertaken non‐promotional educational and/or consultancy work for Abbott UK, Insulet, Dexcom, Sanofi, Lilly, and Roche; Sufyan Hussain's institution has received research grant support from Abbott and Insulet. Tadej Battelino is an IDF Europe Board Member and has served on advisory panels of Novo Nordisk, Sanofi, Eli Lilly, Astra Zeneca, Medtronic, Abbott, Pfizer, Tandem, and Roche. Tadej Battelino received honoraria for participating on the speaker's bureaux of Eli Lilly, Novo Nordisk, Medtronic, Abbott, Sanofi, Dexcom, Aventis, Astra Zeneca, and Roche. Tadej Battelino's Institution received research grant support from Abbott, Medtronic, Novo Nordisk, Sanofi, Novartis, Sandoz, and Tandem, Slovenian Research and Innovation Agency, the National Institutes of Health, BreakthroughT1D, Helmsley Foundation, and the European Union. All other authors report no conflicts of interest.

## Data Availability

Data available on request from the authors.
